# Pearl Grey Shading Net Boosts the Accumulation of Total Carotenoids and Phenolic Compounds That Accentuate the Antioxidant Activity of Processing Tomato

**DOI:** 10.3390/antiox10121999

**Published:** 2021-12-15

**Authors:** Luigi Formisano, Michele Ciriello, Christophe El-Nakhel, Milena Poledica, Giuseppe Starace, Giulia Graziani, Alberto Ritieni, Stefania De Pascale, Youssef Rouphael

**Affiliations:** 1 Department of Agricultural Sciences, University of Naples Federico II, 80055 Portici, Italy; luigi.formisano3@unina.it (L.F.); michele.ciriello@unina.it (M.C.); christophe.elnakhel@unina.it (C.E.-N.); youssef.rouphael@unina.it (Y.R.); 2 Research and Development Lab, Sachim S.r.l., 70017 Putignano, Italy; milena.poledica@sachim.it; 3 Department of Management, Finance and Technology, University of Bari LUM Giuseppe Degennaro, 70010 Casamassima, Italy; starace@lum.it; 4 Department of Pharmacy, University of Naples Federico II, Via Domenico Montesano 49, 80141 Napoli, Italy; giulia.graziani@unina.it (G.G.); ritialb@unina.it (A.R.)

**Keywords:** *Solanum lycopersicum* L., shading screens, industrial tomato, UHPLC/HRMS, HPLC-DAD, lycopene, chlorogenic acid, rutin, FRAP, ABTS

## Abstract

Tomato (*Solanum lycopersicum* L.) is one of the most consumed vegetables worldwide due to its low caloric intake and high fiber, minerals, and phenolic compounds, making it a high-quality functional food. However, fruit quality attributes can be affected by pre-harvest factors, especially environmental stresses. This research aimed to evaluate the influence of two shading nets (white net −30% and pearl grey net −40% shading degree) on the yield and phytochemical profile of tomato fruits grown in summer under the Mediterranean climate. Mineral and organic acid content (by ion chromatography-IC), phenolic profile (by ultra-high performance liquid chromatography-UHPLC coupled with an Orbitrap high-resolution mass spectrometry-HRMS), carotenoid content (by high-performance liquid chromatography with diode array detection-HPLC-DAD), and antioxidant activities DPPH, ABTS, and FRAP (by UV-VIS spectrophotometry) were determined. Tomato fruits grown under the pearl grey net recorded the highest values of total phenolic compounds (14,997 µg 100 g^−1^ of fresh weight) and antioxidant activities DPPH, ABTS, and FRAP, without affecting either fruit color or marketable yield. The reduction of solar radiation through pearl grey nets proved to be an excellent tool to increase the phytochemical quality of tomato fruits during summer cultivation in a Mediterranean environment.

## 1. Introduction

Providing a comprehensive definition of vegetable quality nowadays is an ever-increasing meticulous task. Historically, the primary goal of the horticultural supply chain was to ensure food security by breeding ‘high yielding’ genotypes, considering quality as something exclusively related to visual attributes such as size, shape, and color [1,2,3]. However, a changed socio-economic and cultural context have accelerated the transition to a ‘consumer-oriented’ model, where the consumer is aware and informed about the nutraceutical value of vegetables [1,4]. The irreconcilable contrast between the frenetic rhythms imposed by modern times and the desire for a healthy lifestyle has drawn attention to the importance of a nourishing diet as a lifeline [5]. A healthy lifestyle diet based on the consumption of large portions of fruits and vegetables, such as the Mediterranean diet, is a powerful weapon for reducing the incidence of pathological disorders through a regular intake of natural boosters such as vitamins, minerals, and phytonutrients [6].

Native to South America, tomato (*Solanum lycopersicum* L.) is a staple food of healthy dietary regimen, as well as an essential raw ingredient of recipes and processed products, appointing it among the most consumed foods worldwide [3,7,8]. The premium-quality organoleptic properties of ripe tomato fruits are due to the interaction of soluble sugars (glucose, fructose, and sucrose) and organic acids (citric and malate), which give a perfect mix of sweetness, acidity, and tastiness [2,3,9]. The intense red coloration, known to influence consumer perceptions, is attributed to lycopene, the most abundant carotenoid and accounting for about 80% of the total pigments [10,11]. As a determinant of the visual quality of fruits, lycopene is known to be beneficial for human health, since carotenoids cannot be synthesized ex novo by humans, but must necessarily be introduced through the diet [3,12]. A recognized beneficial action exerted by lycopene, related to its antioxidant activity, has been highlighted in several studies showing a negative correlation between its intake and the incidence of chronic diseases [10,13]. However, it is worth noting that tomatoes also contain other pigments such as *α*-carotene, *β*-carotene, and lutein, which contribute equally to the nutritional value [3]. The low caloric value and well-recognized benefits of carotenoids complete the richness in fiber, minerals, and phenolic compounds of tomatoes, making it an excellent functional food [7]. In addition, Slimestada and Verheulb [14] reported about 100 phenolic compounds in tomatoes, of which the most abundant flavonoids are quercetin and kaempferol derivatives (rutin and naringenin), while for phenolic acids it is chlorogenic acid [12,15]. The high bioactivity of phenolic compounds bestows them potent antioxidant activities that can trigger anti-inflammatory, anti-atherogenic, anti-tumor, hepatoprotective, antiviral, and cardioprotective responses; attributes that are increasingly desired in foods [2,16].

It is well established in literature that most quality traits of tomatoes can shift according to preharvest factors, such as genotype, harvest, and ripening stage, growing conditions, and especially environmental stresses [3,11,17]. Wang et al. [18] documented that microclimatic factors, remarkably, light and temperature, affect the phytochemical profile of fresh horticultural products, resulting in an ongoing modification of their nutritional quality. However, many authors agree that environmental factors which are most likely to affect the nutritional value of tomatoes are temperature and light [3,15,19,20]. Pressman et al. [21] and Sato et al. [22] showed that yield parameters (number and weight of fruits) are negatively affected by average temperatures above 29 °C due to pollination or fruit set defects. Moreover, Spicher et al. [23] and Lu et al. [24] did not detect structural damage to the photosynthetic apparatus during the vegetative development stage at temperatures near 38 °C.

Light is an essential abiotic component for plant growth, as it provides energy for photosynthesis and is crucial for many physiological processes and qualitative aspects [25]. Quality traits of tomato fruits, such as the content of vitamin C, carotenoids, and phenols, are firmly conditioned by light intensity and duration [3]. The available literature review shows that the phytochemical content of tomato fruits under high light radiation is ambiguous. For example, tomatoes grown under high light intensity led to high flavonoid content, probably due to increased UV-B radiation [3]. Conversely, it has been reported that high light intensity can impair lycopene accumulation with repercussions on intrinsic quality attributes of tomato fruits [11,26].

In Mediterranean areas, shading nets are extensively used for reducing solar radiation in summer crop cycles, thereby minimizing the occurrence of cracking and discoloration in tomato fruits as they provide a mixture of diffuse and unmodified natural light from which plants benefit [19,27]. Solar radiation is the main parameter influenced by the shading nets and depends on the design properties such as the number of meshes per cm and the shade factor [19,28,29,30]. The aim of our work was to evaluate the influence of shading nets on the yield and particularly on the phytochemical profile of tomato fruits grown in midsummer in a Mediterranean climate. For this purpose, two different shading nets with varying shading factors were used (white net: 30% shading factor; pearl grey net: 40% shading factor), which can be of additional technique and advancement to modulate the qualitative attributes of tomato.

## 2. Materials and Methods

### 2.1. Plant Material, Experimental Design, and Growth Conditions

The trial was conducted in spring-summer 2021 at ‘Raffaele Tamburrino’ farm, located in Villa Literno (Caserta, Italy, 10 m above sea level). Tomato (*Solanum lycopersicum* L.) Quorum F1 seedlings (ISI sementi S.p.A., Fidenza, Italy) were transplanted at the phenological stage of three true-leaves on 3 June 2021, arranged in a double row at a density of 3.5 plants per m^2^. The experimental protocol was based on comparing two shading nets supplied by Arrigoni S.p.A (Uggiate Trevano, Como, Italy) plus an unshaded control, each corresponding to a plot of 240 m^2^ (experimental unit) which was randomized in three replicates. Net characteristics were as follows: (1) 2633BL Prism LDF (hereafter ‘white net’; shading factor: 30%; air permeability: 44%); (2) 2633GP Prism LDF (hereafter ‘pearl grey net’; shading factor: 40%; air permeability: 44%). Fertilization, irrigation, and control of phytopathogens were carried out according to the standard agricultural practices of the cultivation area. Specifically, one month before transplanting, disc harrowing and soil leveling were performed. Water was supplied through a drip irrigation system every two or three days. Nutrient management was performed by fertigation with 150 kg ha^−1^ of N, 40 kg ha^−1^ of P_2_O_5_, and 220 kg ha^−1^ of K_2_O. Phosphorus was entirely supplied during soil preparation operations (pre-transplanting), while nitrogen and potassium were supplied before crop establishment (30% and 55% for N and K_2_O, respectively) and the remainder during the crop cycle. The crop was protected against *Phytophthora infestans*, *Tuta absoluta*, *Aphids* spp., *Bemisia tabaci*, *Trialeurodes vaporariorum*, and *Tetranychus urticae*. Climatic parameters, such as relative humidity, air temperature, and photosynthetically active radiation (PAR), were continuously recorded using WatchDog A150 dataloggers (Spectrum Technologies Inc., Aurora, IL, USA; ± 0.6 °C/±3% Temp/RH accuracy) placed at 0.5 m above ground level. During the experiment, the average air temperature was 28.1 °C, 27.4 °C, and 26.8 °C for the white net, pearl net, and control, respectively.

### 2.2. Fruit Harvest, Yield, and Fruit Quality Measurement

The experimental trial lasted a total of 91 days (3 June to 1 September). At harvest (91 days after transplanting, DAT), fruits of 15 representative plants were sampled for each replicate, avoiding border plants. The fruits were counted, weighed, and separated into two groups: marketable (ripe and free of visible defects) and unmarketable (misshapen, undersized, and green) fruits. The equatorial and polar diameters were determined on the marketable fruits using digital caliper (±0.02 mm accuracy; RS PRO, Sesto San Giovanni, Milan, Italy). A representative sample of the marketable fruits was blended in a Waring^®^ blender (2 L capacity; Model HGB140, McConnellsburg, PA, USA) for 1 min and filtered to determine the juice quality. From the extracted juice, the total soluble solids (TSS) content, expressed as °Brix at 20 °C, was determined using an Atago N1 portable digital refractometer (Atago Co. Ltd., Tokyo, Japan). An aliquot of fruit juice (approximately 100 g) was dried in a ventilated oven at 70 °C until a constant weight was reached to determine the percentage of dry matter. The dried fruit material was then blended with a KM13 rotating blade grinder (Bosch, Gerlingen, Germany) and stored for mineral and organic acid analysis.

A part of the marketable fruits was immediately frozen at −80 °C and underwent a freeze-drying cycle (Alpha 1–4 Martin Christ Gefriertrocknungsanlagen GmbH, Osterode am Harz, Germany) for further qualitative analysis.

### 2.3. Determination of Fruit Color Using CIELab Color Space

Twenty marketable fruits per replicate were selected to determine colorimetric indices using a Minolta Chromameter CR-400 portable colorimeter (Minolta Camera Co. Ltd., Osaka, Japan). For each fruit, two colorimetric measurements were made (on two opposite sides of the fruit) of the indices L (brightness, 0 to 100), a* (greenness, −60 to +60), and b* (yellowness, −60 to +60). Chroma (‘colorfulness’ quantitative attribute) and Hue angle (qualitative color attribute in the relative amounts of redness and yellowness) were calculated as described by the International Commission of Illumination (CIE):

Chroma = [(a*)^2^ + (b*)^2^]^0.5^

Hue angle = tan^−1^ b*/a*

### 2.4. Mineral Content Determination

The determination of cations (K, Mg, and Na), anion (P), and organic acids (malate and citrate) was carried out by ion chromatography according to the protocol described in detail by Formisano et al. [31]. Briefly, 250 mg of dried and finely ground fruits were mixed with 50 mL of ultrapure water, extracted for 10 min in a water bath at 80 °C, and then centrifuged at 6000 rpm for 10 min. Twenty-five μL of the supernatant, filtered through a 0.45 μm syringe filter, was injected into an ion chromatographic system coupled with an electrical conductivity detector (ICS 3000, Thermo Scientific^TM^ Dionex^TM^, Sunnyvale, CA, USA). The isocratic separation of the cations was performed using 25 mM methanesulfonic acid as eluent (Sigma Aldrich, Milan, Italy) using an analytical column IonPac^®^ CS12A (4 × 250 mm) equipped with an IonPac^®^ CG12A precolumn (4 × 250 mm) and a CERS500 autoregenerating suppressor. The separation of organic acids and the anion P was carried out in gradient mode with potassium hydroxide (5 mM-30 mM, flow rate of 1.5 mL min^−1^) using an IonPac^®^ ATC-HC anion trap (9 × 75 mm), an IonPac^®^ AG11-HC guard column (4 × 50 mm), an IonPac^®^ AG11-HC IC column (4 × 50 mm), and a DRS600 auto-regenerating dynamic suppressor. All analytical columns, precolumns, traps, and suppressors were purchased from Thermo Scientific^TM^ Dionex^TM^ (Sunnyvale, CA, USA). The concentrations of the minerals and organic acids in fruits were expressed as mg 100 g^−1^ of fresh weight (fw). Each treatment was analyzed in triplicate.

### 2.5. Determination of the Polyphenol Profile by Ultra-High Performance Liquid Chromatography (UHPLC) and Orbitrap High-Resolution Mass Spectrometry (HRMS) Analysis

Polyphenols profile detection and quantification were performed according to the protocol described in detail by El-Nakhel et al. [32]. Briefly, 5 μL of the extracted samples according to the procedure described by Vallverdú-Queralt et al. [33], were analyzed using a Dionex Ultimate 3000 ultra-high-pressure liquid chromatography (UHPLC) system (Thermo Fisher Scientific^TM^, Waltham, MA, USA) coupled to an Orbitrap high resolution mass spectrometry (HRMS) (Thermo Fisher Scientific^TM^, Waltham, MA, USA). The chromatographic separation of polyphenols was carried out with a Luna Omega PS (1.6 μm, 50 × 2.1 mm, Phenomenex, Torrance, CA, USA) thermostated column (T = 25 °C). The mobile phase consisted of a two-phase solution: water (phase A) and acetonitrile (phase B). Both mobile phases contained 0.1% formic acid (*v*/*v*). An ESI source (Thermo Fisher Scientific^TM^, Waltham, MA, USA) was used in negative ion mode (ESI–), setting two scan events (Full ion MS and All ion fragmentation, AIF) for all compounds of interest. Data processing was performed with Quan/Qual Browser Xcalibur software, v. 3.1.66.10 (Thermo Fisher Scientific^TM^, Waltham, MA, USA). Polyphenols were expressed as μg 100 g^−1^ fw.

### 2.6. Spectrophotometric Determination of ABTS, DPPH, and FRAP Antioxidant Activities

The ABTS^+^ antioxidant activity was performed as described by Re et al. [34]. The solution of 2,2′-azinobis-(3-ethylbenzothiazoline-6-sulphonate radical (ABTS^+^) in water was obtained using the classical method of ABTS incubation in darkness at 23 °C for 16 h with potassium peroxydisulfate. After incubation, the stock solution was diluted with ethanol (1:88) until reaching an absorbance of 0.700 ± 0.050 at 734 nm. A 0.1 mL aliquot of each sample that was previously filtered and diluted (1:10) with 70% methanol, was mixed with 1 mL of ABTS^+^ solution and stored at ambient temperature for 2.5 min. The absorbance was immediately recorded at 734 nm.

The radical-scavenging activity of 2,2-diphenyl-1-picrylhydrazyl (DPPH) was determined according to the protocol proposed by Brand-Williams et al. [35]. A 1 mL aliquot of DPPH solution (4 mg 10 mL^−1^ of 96% methanol) was added to 200 μL of the studied extract, mixed, and incubated at ambient temperature for 10 min. The absorbance was recorded at 517 nm.

The determination of the ferric reduction antioxidant power (FRAP) assay was performed following the protocol described by Rajurkar and Hande [36] with minor modifications. This assay is based on the fact that antioxidants reduce ferric ions to ferrous ions, creating a blue complex (Fe^2+^/2,4,6-tris(2-pyridyl)-s-triazine, TPTZ) with an absorption peak at 593 nm. Briefly, 150 μL of each sample was mixed with 2.850 mL of FRAP solution (1.25 mL of 10 mM TPTZ solution in 40 mM HCl + 1.25 mL of 20 mM FeCl_3_ in water + 12.5 mL of 0.3 M acetate buffer, pH 3.6) and incubated for 4 min. The absorbance at 593 nm was then read.

The absorbances of the ABTS, DPPH, and FRAP assays were recorded by UV-VIS spectrophotometer (Shimadzu, Japan). The results were expressed as mmol Trolox equivalents kg^−1^ dw. All analyses were performed in triplicate.

### 2.7. Carotenoids Determination

Carotenoids were quantified by high-performance liquid chromatography with diode array detection (HPLC-DAD) according to the protocol of Salomon et al. [37]. Briefly, 0.1 g of lyophilized tissue was macerated with 1 mL of ultra-pure water and 5 mL of ethanol/n-hexane (60:50, *v*/*v*) and then sonicated and centrifuged (15 min at 4000 rpm). After removing the solvent phase by vacuum dry centrifugation, the pellet was subjected to two vacuum extraction/centrifugation cycles. A mixture of methanol and methyl-t-butyl ether (MTBE) (1:1, *v*/*v*) was added to the completely dried pellet and analyzed by the HPLC-DAD technique. Calibration curves were constructed using commercial *β*-carotene and lutein standards purchased from Sigma-Aldrich (Milan, Italy). Results were expressed as mg 100 g^−1^ fw. All analyses were performed in triplicate.

The lycopene content of the fruits was determined by spectrophotometry according to the protocol described by Sadler et al. [38]. Lycopene quantification was performed by measuring the absorbance of the hexane extract at 472 nm, using pure lycopene (Sigma-Aldrich, Milan, Italy) to construct the calibration curve. Lycopene content was expressed as mg 100 g^−1^ fw. All analyses were performed in triplicate.

### 2.8. Statistical Analysis

All data were analyzed with IBM SPSS Statistics software (SPSS Inc., Chicago, IL, USA) version 26.0 for Windows 10 and are presented as mean ± standard error, *n* = 3. All mean effects were subjected to one-way ANOVA analysis. Statistical significance was determined with Tukey’s HSD test at the *p* = 0.05 level. All plant responses to shading treatments were summarized via a color heatmap generated using the web tool ClustVis (https://biit.cs.ut.ee/clustvis/, accessed on 9 December 2021). The Euclidean distance was used as a measure of similarity and hierarchical clustering with complete linkage heatmaps, and the data were normalized and visualized using a false color scale (red = increase in values; blue = decrease in values) [39].

## 3. Results and Discussion

### 3.1. Microclimatic Parameters

Shading nets significantly reduced PAR compared to the unshaded control (Table 1). In June, the mean PAR in the open field (control) was 1247 μmol m^−2^ s^−1^ in contrast to the mean PAR observed under white and grey shading nets with a mean value of 871 and 703 μmol m^−2^ s^−1^, respectively. In July, the mean PAR of the control was 1271 μmol m^−2^ s^−1^, approximately 2.0% higher than in June. In August, the mean PAR was the lowest, with the control averaging 1127 μmol m^−2^ s^−1^ (Table 1).

In the present study, the effect of the temperature was separated from that of the solar radiation (Table 1 and Table 2). The highest mean temperature was recorded in July under the white net (29.3 °C), while the lowest was recorded in June under the pearl grey net (26.8 °C). However, regardless of the mean PAR values and the degree of shading, the difference in temperature recorded outside and under the shade nets was not significant (Table 2).

### 3.2. Yield and Yield Parameters

Tomato is one of the most consumed vegetables worldwide and represents one of the driving crops for many countries economy, due to its dual use as a fresh and processed product (e.g., pasta, sauce, peeled tomatoes, juice, ketchup) [8,10]. To date, world tomato production is estimated at 180 million tons, with China alone having a total production of approximately 63 million tons, followed by India (~19 million tons), Turkey (~13 million tons), the United States of America (~11 million tons), Egypt (~7 million tons), and Italy (~5 million tons) [40]. However, it is well known that tomato yield is strongly influenced by environmental factors (such as humidity, temperature, and solar radiation), genotype, and pre-harvest factors (growing practices) [19,41].

In our study, the number of total fruits per plant showed a significant decrease compared to the control when the plants were under shading (Control > White net > Pearl grey net; Table 3). Probably, high light intensity conditions induced an eco-physiological response to mitigate stress, increasing the number of fruits but reducing the transpiring surface (diameter of the fruit) compared to shaded conditions. Consequently, the reduction in the fruit number induced by shading did not affect the weight of the fruit (Table 3). Total, marketable, and unmarketable fruits weight (kg pl^−1^) did not show significant differences in shaded plants compared to the control, in contrast to the findings of Angmo et al. [42], who reported an increase in total marketable fruit weight in the open field compared to shaded conditions, which could be attributed to different environmental conditions, genetic material, and cultural practices [43,44,45]. In the present experiment, we adopted a processing tomato cultivar that was neither tied nor defoliated, in contrast to the methods used by the authors mentioned above, and in part, could have determined different production responses. Shading nets resulted in a considerable increase in marketable fruit weight of 47.5%, compared to the control, justifying the non-significant difference in total fruit weight per plant (Table 3).

### 3.3. Quality Attributes of Fruits

The growing interest in high-quality food products has forced growers to meet the changing needs of increasingly demanding consumers. In the past, the marketable quality of vegetables relied primarily on visible characteristics, but now sensory and organoleptic characteristics have become a primary parameter driving consumer choice [2,46]. In tomatoes, one of the sensory attributes that determine the organoleptic quality of the fruit is the content of soluble solids (glucose, fructose, and sucrose), which, combined with organic acids and amino acids, represents approximately 75% of dry matter [46]. In the literature, it is known that fruit sweetness is strongly influenced by genetic material [26]. Almeida et al. [20] have studied the effects on the accumulation of total soluble solids (TSS) of five genotypes of tomatoes under different environmental conditions. The authors found that the TSS content ranged among the genotypes from 5.6 to 7.2 °Brix, and according to the environmental conditions from 3.8 to 8.9 °Brix. In our experiment, we found that light radiation affected this crucial qualitative parameter (Table 4). Davies et al. [47] highlighted the evidence of a direct relationship between solar radiation and sugar content in tomatoes. Our results confirm this correlation as fruits exposed to direct solar radiation (Control) showed the highest value of TSS (7.43 °Brix; Table 4), confirming what was reported by Ilić et al. [48] in a similar experiment. The higher TSS content in the control fruits was probably attributable to the lower water assimilation capacity of the fruits, which also justified the high dry matter content (8.71%; Table 4) [49]. However, it is worth considering that an increase in the amount of solar radiation received by the plant may increase photosynthesis, and thus carbohydrates in the fruit [8]. On the other hand, the direct correlation between solar radiation and TSS is not univocal, as different results are found in the literature, again highlighting how genotype plays a crucial role in the adaptation to different environmental conditions [10,48].

Another qualitative aspect that can influence consumer choice is color, since a well-colored fruit is qualitatively superior. Practically, the color of the fruit depends on the physical and biochemical changes that occur naturally during the growth and ripening stages or after harvest [50]. Among the CIELab colorimetric parameters, only the L (brightness) parameter varied significantly in response to shading, with the highest value obtained in fruits grown under white shading nets (Table 4). For definition, L is “an approximate measure of brightness, which is the property according to which any color can be considered equivalent to a member of the greyscale, between black and white” [51]. The increase in L under shaded conditions agrees with the findings of Messina et al. [52]. However, the same authors also reported a decrease in a* values (less intense red color) and an increase in b* values (more intense yellow color) that we did not observe in our study (Table 4).

Similar to TSS content and color, fruit size and shape are also essential quality traits. Although shape is primarily determined by genetic background, size also depends on the interaction of the latter with the environment [2]. From a physiological point of view, the increase in fruit size depends on the enlargement of the pericarp due to the production of new cells during the anthesis process and the growth and expansion of cells that last until the fruit ripening [2]. According to Angmo et al. [42], compared to the control, we observed an average increase in the equatorial and polar diameter of fruits of 8.60 and 10.50%, respectively, when grown under shading nets (Table 4). The larger fruit size recorded under shading nets accounted for the higher average marketable fruit weight (Table 3), attributed to the higher water content in the fruit (lower dry matter) that resulted in a dilution effect on TSS (Table 4).

### 3.4. Mineral Content of Fruits

Minerals, like other macromolecules (carbohydrates, proteins, and fats), are required to preserve some physical and biochemical processes essential for life [53]. Currently, mineral deficiency in the human diet is a severe problem for industrial and developing countries [54]. Given the high intake of tomatoes, the potential contribution of tomato fruit to the mineral intake of human diet is of high importance [55]. It is well established that the most abundant mineral in tomato fruit is potassium [54]. Potassium plays a crucial role in maintaining cellular homeostasis, nerve impulse conduction and muscle contraction, and the glycogenesis process [53,56]. In plants, potassium is an activator of enzymatic processes and contributes significantly to the photosynthetic process [57]. Among the macronutrients reported in Table 5, potassium was the most abundant mineral in the fruit and was affected by shading treatment, with the highest value recorded under white net. Although potassium is crucial for color determination, the change in its content was not coupled with a perceived change in color (a*) (Table 4). This result could be partially related to the optimal potassium content (361.43–445.65 mg 100 g^−1^ fw) [57].

Phosphorus is the main component of bones, and is involved in many metabolic processes (kidney function and cell growth); it has a buffering action and is involved in the formation of high-energy compounds (adenosine triphosphate) and in phospholipid synthesis [53,58]. Similarly to potassium, the phosphorus content was significantly affected by growth conditions (Table 5). The higher value (14.88 mg 100 g^−1^ fw) recorded in fruits grown under unshaded conditions would help to better explain the higher TSS obtained from the same treatment. Indeed, Lavon et al. [59] showed a positive correlation between this essential macroelement and TSS content in tomato fruits.

Although magnesium deficiency in the human diet is rarely a determinant of pathological states (WHO; [60]), this mineral is crucial, as it is a component of bones and teeth and is an active component of different enzymatic systems [53]. In our study, the magnesium content was not affected by shading (Table 5). Although Milenković et al. [19] observed a reduction in magnesium in tomato fruits exposed to direct solar radiation, our results do not show the same trend. This discordance could be attributable not only to the different genetic material, but also to the different light conditions.

Tomato acidity is a crucial component of the organoleptic quality of fruits [61]. Interactions between reducing sugars and organic acids are essential to confer sweetness, tartness, and flavor intensity to fruits [2,3,9]. The main organic acids in tomato fruits are malic and citric acids, but the perception of acidity is mainly due to the latter, which is the most abundant organic acid [2,61]. Shading treatments reduced the citrate content in fruits by 23.7%, compared to the control that showed the highest values (140.36 mg 100 g^−1^ fw). The higher citrate content in the fruits of the control could probably be attributable to a higher source:sink ratio during the pre-ripening phase, increasing the respiration rate of the fruits. Therefore, a higher respiration rate could have promoted glycolysis and increased citrate production [61]. However, it should be noted that changes in fruit water content were observed between treatments (Table 3), which may have interfered with acidity due to a dilution/dehydration effect [61].

### 3.5. Fruit Pigments

The relevance of the quanti-qualitative profile of carotenoids in tomato fruits is mainly attributable to their dual function. In fact, while these biomolecules determine the coloration of ripe fruits, they are crucial in the human diet due to their recognized antioxidant activity [2,62]. Furthermore, it is important to note that the body cannot synthesize these valuable pigments, making their intake through plant consumption mandatory [63]. In plants, carotenoids are used to capture light and protect the photosynthetic apparatus from excessive solar radiation, attract pollinators, and facilitate seed dispersal [2,62]. The data reported in Table 6 show that, compared to the control, shading treatments resulted in the highest biosynthesis of total carotenoids in fruits. Although there is a wide variability in carotenoid content in the literature, our results are in agreement with the findings of Flores et al. [64] in red tomato fruits, which showed that the most abundant carotenoid was lycopene, followed by *β*-carotene, and lutein. Compared to shaded conditions, the ~40.0% reduction in the lycopene content in control fruits confirms that excessive radiation exerts an inhibitory effect on the biosynthesis and accumulation of this critical pigment [3,65,66]. Leyva et al. [3] noted that the decrease in lycopene content which is found in our work as well, could be attributed to direct solar radiation and not air temperature, since Helyes et al. [67] observed that fruit surface temperatures of 30 °C trigger the degradation of this pigment. These conditions could have occurred in our case under control unshaded conditions.

Lycopene is a crucial intermediate in the biosynthesis of many carotenoids such as *β*-carotene and xanthophylls such as lutein [63]. Consequently, it is not surprising that high solar radiation (Control) resulted in an average reduction in *β*-carotene (−43.4%) content, compared to shaded conditions. Not least, it is interesting to note that although lycopene is responsible for the red color of tomatoes [68], the significant differences in lycopene content between treatments did not affect the colorimetric parameter a* (Table 4). This result could be related to the direct correlation between lycopene content and fruit size (equatorial diameter and polar diameter; Table 4), which probably influenced the colorimetric analyses.

### 3.6. Phenolic Compounds and Antioxidant Activity of Fruits

In their natural habitats, plants are threatened by a large number of potential enemies, and to defend themselves, they produce a wide range of heterogeneous protection compounds (pigments, signaling molecules, and aromas) known as ‘secondary metabolites’, which play an important role in their survival [69]. Secondary metabolites are classified on the basis of their chemical structure and biosynthetic pathways. They can be divided into three groups: terpenoids, phenolic compounds and flavonoids, and sulfur-containing compounds and nitrogen-containing alkaloids [70]. Present in most fruits and vegetables, secondary metabolites show beneficial effects on human health [71]. They have well-established anticancer, antiaging, anti-diabetic, and anti-obesity activity, in addition to their protection against Alzheimer’s and cardiovascular diseases [70]. Synthesized through the shikimate biochemical pathway, phenolic compounds represent the most abundant type of secondary metabolites in plants [72]. Their biosynthesis begins from non-oxidative deamination of phenylalanine, mediated by the enzyme phenylalanine ammonia-lyase (PAL), leading to the formation of cinnamic trans acid as a key intermediate at the base of secondary products derived from phenylpropanoid (flavonoids and isoflavonoids, coumarins, lignins, esters of hydroxycinnamic acid, and phenolic compounds) [72]. The accumulation of these compounds varies between organisms, tissues, and growth stage, and can be influenced by environmental conditions, because gene expression levels that encode key enzymes in the phenylpropanoid biosynthesis pathway are affected by environmental stressors (light, temperature, and nutritional deficits) [73].

The UHPLC analysis identified 20 phenolic compounds that could be classified into the following categories: phenolic acid derivatives, flavonoid derivatives, and hydroxycinnamoyl quinic acid derivatives (Table 7). The different light intensity conditions that characterized the treatments in the present experiment influenced the total content of phenolic compounds, calculated as the sum of all the detected individual phenolic compounds. Specifically, the Pearl grey shade net resulted in the highest accumulation of total phenolic compounds (14,997 µg 100 g^−1^ fw), followed by the control (12,377 µg 100 g^−1^ fw) and the White shade net (9869 µg 100 g^−1^ fw). Regardless of the treatment, the largest contribution to total phenolic compounds resulted from flavonoid derivatives (7776 µg 100 g^−1^ fw, on average), as reported by Bertin and Génard [2], followed by phenolic acid derivatives (4097 µg 100 g^−1^ fw, on average) and lastly hydroxycinnamoyl quinic acid derivatives (541 µg 100 g^−1^ fw, on average). Although flavonoids are ‘semi-essential’ compounds, having no well-defined nutritional function, they are crucial for protecting antioxidant compounds from oxidative degradation in humans and plants [74].

As reported by Slimestad and Verheul [8] and Abreu et al. [75], regardless of treatments, rutin was the most abundant flavonoid (Table 7). In contrast, Bertin and Génard [2] reported that the most abundant flavonoid in tomatoes was naringenin, demonstrating how genotype, cultural practices, environmental conditions, and even analytical determination techniques can influence the content of these compounds. Rutin is considered one of the best natural antioxidants currently known that can exert important pharmacological activities, acting as antibacterial, anti-inflammatory, antiallergic, antiviral, antiprotozoal, and antitumor. It has also marked cytoprotective, vasoactive, antiplatelet, hypolipidemic, and antihypertensive activities [76]. The highest rutin values were obtained using a Pearl grey shading net (4414 µg 100 g^−1^ fw), similarly to the other flavonoid derivatives (kampferol-3-diglucoside > naringenin > rutin-O-pentoside > kaempferol-3-O-rutinoside > naringenin-C-diglycoside > apigenin-C-hexoside-hexoside > naringenin-C-hexoside > quercetin-O-dihexoside > genistin), except for rutin-O-hexoside, which showed the highest value under the White shading net and control (Table 7). The reviewed literature shows that the increase in flavonoids (mainly rutin) is promoted by intense solar radiation [77,78], which is not in line with the results obtained in this experiment. The total flavonoid content of fruits grown under pearl grey net was 59.9% higher than that recorded in the unshaded control (Table 7). The reason behind this can be partly explained by the fact that most of the reviewed works did not separate the effect of temperature from that of solar radiation, while in our study, the only significantly different parameter was PAR (Table 1 and Table 2). Furthermore, it should be considered that the response of the plant to a stressor depends not only on the genotype, intensity, and magnitude of the stressor, but also on the stage of development of the plant and the organs involved [2]. For example, despite evidence in the literature that water stress can increase the levels of phenolic compounds, Atkinson et al. [79] observed that the greatest accumulation of flavonoids was recorded in tomato leaves following water stress, while in fruits no changes were observed. Similarly, Abreu et al. [75] showed, in line with our results, that shading increased the phenolic content in tomato fruits, compared to the control, that resulted in a greater accumulation of these compounds only in the leaves. This result was probably attributable to the fact that the leaves were directly exposed to the stressor (high direct solar radiation). Not least, the obtained up-regulation of flavonoids could result not only from the limitation of solar radiation recorded in August under the pearl grey net (Table 1), but also from eco-physiological responses induced by the different microclimatic conditions that lead to the plant to modify its primary metabolism (fewer but larger fruits; Table 3). As argued by Campa et al. [80], low light intensity would have triggered the production of phenolic compounds with which plants would have counterbalanced the reduction in antioxidant enzyme activity. In contrast with flavonoids trend, the highest hydroxycinnamoyl quinic acid derivatives values were obtained under Pearl grey net (685 µg 100 g^−1^ fw) and in the control (633 µg 100 g^−1^ fw), while the lowest were obtained under White net (305 µg 100 g^−1^ fw) (Table 7). In particular, the values of tricaffeoylquinic and dicaffeoylquinic acids in the more shaded conditions (pearl grey) were 53.30 and 44.50% higher than the average of the other treatments.

In contrast to what was observed for flavonoid derivatives and hydroxycinnamoyl quinic acids, the content of phenolic acid derivatives was lower in fruits harvested under pearl grey shading net (Table 7). As reported in the literature, chlorogenic acid is the main non-flavonoid phenolic compound found in tomato fruits that possess high antioxidant, antibacterial, anti-inflammatory, antiviral, antimicrobial, hepatoprotective, cardioprotective, and neuroprotective properties [2,8,81,82]. The highest value of this crucial antioxidant compound was found in the unshaded control (3363 µg 100 g^−1^ fw) while the lowest was found in the Pearl grey net treatment (1,799 µg 100 g^−1^ fw) (Table 7). However, Botella et al. [83] reported that homovanillic acid-O-hexoside was the primary compound in tomato fruits. In our case, homovanillic acid O-hexoside was found to be the second most prevalent compound, with the highest values (11.51 µg g^−1^ fw) recorded in fruits grown under the white net. The lowest values of coumaric acid O-hexoside were found in the White net treatment, whereas the lowest ones of ferulic and caffeic acids were found in the control (Table 7).

In contrast to what was observed with flavonoid and hydroxycinnamoyl quinic acid derivatives, the heterogeneity of phenolic acid derivatives among treatments emphasizes that irradiation and/or shading strongly influenced the biosynthesis of this class of phenolic compounds. Probably, different light conditions could have unequivocally influenced the assignment of phenolic substrates to individual branches of the phenylpropanoid pathway [73].

Different spectrophotometric assays were carried out for the determination of antioxidant activity of tomato fruits. Specifically, we evaluated the free radical scavenging activity DPPH, the free radical scavenging activity by ABTS decolorization, and the ferric reducing antioxidant capacity FRAP (Table 8). Our findings showed significant antioxidant activity in fruits grown under pearl grey net, probably related to the higher content of total phenolic compounds (Table 7) [75].

In detail, DPPH antioxidant activity increased as the degree of shading increased, with the highest (40.72 mmol Trolox eq. kg^−1^ dw) and lowest (32.21 mmol Trolox eq. kg^−1^ dw) values obtained in fruits shaded with the pearl grey net and in the control, respectively. On the contrary, the ABTS assay did not show the same trend, with the lowest value (35.33 mmol Trolox eq. kg^−1^ dw) obtained in fruits shaded with the white net and the highest value (43.70 mmol Trolox eq. kg^−1^ dw) obtained with the pearl grey net.

The FRAP activity showed the highest value (34.38 mmol Trolox eq. kg^−1^ dw) in fruits shaded with the pearl grey net, while no significant differences were observed between the unshaded control and the White net treatment.

Furthermore, the correlation coefficient between the content of total phenolic compounds and antioxidant activities was highly significant, especially for the FRAP (R^2^ = 0.74) and ABTS (R^2^ = 0.99) assays. The correlation coefficient between the DPPH assay and the total phenolic compounds (R^2^ = 0.37) was less significant than that of the FRAP and ABTS assays (Table 8). These discrepancies could be due to synergistic effects between phenolic compounds and other chemical components that can contribute to the total antioxidant activity or the type of assay used [84,85]. The lower value of DPPH found in control plants (Table 8) could be attributed to the lower content of total carotenoids, especially lycopene (Table 6).

### 3.7. Cluster Heatmap of Yield and Quality Parameters of Fruits

A heatmap was performed for all the above parameters to provide a detailed overview of the yield parameters, minerals, pigments, and antioxidant activity of tomato fruits under different shading treatments.

**Figure 1:**
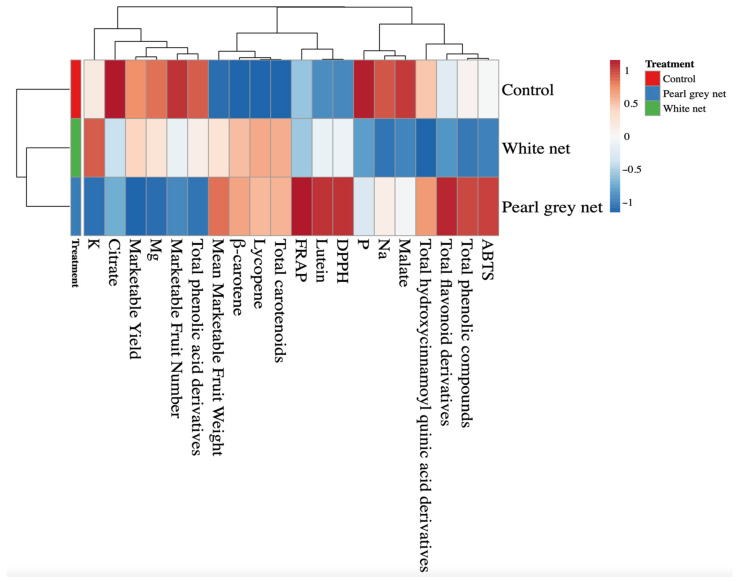
Heatmap analysis summarizing the results of yield, mineral, and quality parameters of *Solanum lycopersicum* L. fruits grown under different shade treatments (Control, White net, and Pearl grey net).

Heatmap analysis of the aggregated data identified two main clusters corresponding to the control and shaded treatments (White net and Pearl grey net), respectively (Figure 1). The separation between the two clusters was mainly due to crucial carotenoids (such as *β*-carotene and lycopene) and total carotenoids. Two separate sub-clusters (White net and Pearl grey net) were defined under the second cluster indicating that shading was the main clustering factor, while unshaded treatment was the second.

The analyzed parameters were separated into three main clusters, each subdivided into secondary sub-clusters (Figure 1). Clusterization of the analyzed parameters shows that the control improved the citrate, phosphorus, and malate content of the fruits compared to the results observed under the white net and Pearl grey. On the contrary, the control reduced the mean marketable fruit weight and the content of *β*-carotene, lycopene, and total carotenoids. The Pearl grey net treatment was characterized by increased antioxidant capacity, total flavonoids derivatives, and lutein.

## 4. Conclusions

The increasing consumer demand for healthy foods with high nutritional value has prompted researchers and producers to focus on production techniques to ensure high yields and premium quality products. In warm Mediterranean regions, high light and high temperatures pose a challenge to tomato production, especially affecting fruit quality and nutraceutical values. The use of shading nets (white and pearl grey) did not significantly affect the average temperature of the growing environments compared to the open field (control). This result allowed us to separate the effect of temperature from solar radiation and to understand more deeply the influence of light on the quality attributes of tomato fruits. Compared to the control, shading reduced, on average, total fruits (−37.93%) without affecting total yield as a result of higher average fruit weight (+46.76%). The higher shade and the better diffusion of light with the pearl grey net led to a more significant accumulation of all compounds with antioxidant activity. Compared to the control, we observed a 70.96% increase in total carotenoids, mainly due to lycopene (+69.74%), and in the content of total phenolic compounds (+21.17%), with the most outstanding contribution given by rutin (4,414 µg 100 g^−1^ fw), Kampferol-3-diglucoside (3,245 µg 100 g^−1^ fw), and naringenin (1,851 µg 100 g^−1^ fw). Finally, the use of the pearl grey net is a valuable tool for the eco-sustainable production of tomatoes, able to preserve and improve the quality attributes of the fruits. The promising results obtained in this study pave the way for future investigations to evaluate the qualitative responses induced by the pearl grey net in other vegetables.

## Figures and Tables

**Table 1:** Photosynthetically active radiation (PAR) during the growing season outside (Control) and under shading nets.

Treatment	June	July	August
Control	1247 ± 5.49 a	1271 ± 7.02 a	1127 ± 7.54 a
White net	871 ± 6.66 b	889 ± 6.43 b	786 ± 3.38 b
Pearl grey net	703 ± 13.3 c	727 ± 3.18 c	633 ± 3.53 c
Significance	***	***	***

*** significant at *p* ≤ 0.001. Different letters within each column indicate significant differences according to Tukey’s HSD test (*p* = 0.05). All data are expressed as mean ± standard error, *n* = 3.

**Table 2:** Air temperature during the growing season outside (Control) and under shading nets.

Treatment	June	July	August
Control	25.9 ± 0.37	27.5 ± 0.32	26.9 ± 0.94
White net	27.1 ± 0.06	29.3 ± 0.31	28.0 ± 0.23
Pearl grey net	26.8 ± 0.53	28.2 ± 0.74	27.1 ± 0.20
Significance	ns	ns	ns

ns non-significant according to Tukey’s HSD test (*p* = 0.05). All data are expressed as mean ± standard error, *n* = 3.

**Table 3:** Effects of shading nets on yield and yield parameters.

Treatment	Yield			Fruit Number			Mean Marketable Fruits Weight (g)
	Total	Marketable	Unmarketable	Total	Marketable	Unmarketable	
	(kg pl^−1^)	(kg pl^−1^)	(kg pl^−1^)	(n° fruits pl^−1^)	(n° fruits pl^−1^)	(n° fruits pl^−1^)	
Control	2.58 ± 0.15	2.25 ± 0.02	0.33 ± 0.06	351.50 ± 9.41 a	268.11 ± 1.66 a	83.39 ± 2.06 a	8.50 ± 0.75 b
White net	2.56 ± 0.24	2.12 ± 0.08	0.44 ± 0.05	251.47 ± 5.55 b	182.98 ± 0.77 b	68.48 ± 1.96 b	11.70 ± 0.76 ab
Pearl grey net	2.20 ± 0.07	1.89 ± 0.08	0.32 ± 0.04	184.88 ± 4.75 c	141.13 ± 1.09 c	43.75 ± 1.55 c	13.37 ± 0.36 a
Significance	ns	ns	ns	***	***	***	*

ns, *, and *** non-significant or significant at *p* ≤ 0.05 and 0.001, respectively. Different letters within each column indicate significant differences according to Tukey’s HSD test (*p* = 0.05). All data are expressed as mean ± standard error, *n* = 3. Pl = plant.

**Table 4:** Effect of shading nets on total soluble solids (TSS), dry matter, CIELab colorimetric parameters, and fruit size.

Treatment	TSS	Dry Matter	L	a *	b *	Chroma	Hue Angle	Equatorial Diameter	Polar Diameter
	(°Brix)	(%)						(mm)	(mm)
Control	7.43 ± 0.30 a	8.71 ± 0.32 a	36.35 ± 0.12 b	28.80 ± 0.90	23.27 ± 0.64	37.03 ± 0.08 ab	218.94 ± 0.59	24.52 ± 0.21 c	33.16 ± 0.01 b
White net	5.40 ± 0.06 b	7.63 ± 0.14 b	38.12 ± 0.24 a	29.49 ± 0.20	23.50 ± 0.16	37.70 ± 0.22 a	218.55 ± 0.21	26.26 ± 0.11 b	36.55 ± 0.17 a
Pearl grey net	5.30 ± 0.25 b	7.25 ± 0.20 b	37.32 ± 0.26 ab	28.21 ± 0.31	23.76 ± 0.35	36.89 ± 0.03 b	220.11 ± 0.62	27.00 ± 0.04 a	36.71 ± 0.13 a
Significance	**	*	*	ns	ns	*	ns	***	***

ns, *, **, and *** non-significant or significant at *p* ≤ 0.05, 0.01, and 0.001, respectively. Different letters within each column indicate significant differences according to Tukey’s HSD test (*p* = 0.05). All data are expressed as mean ± standard error, *n* = 3.

**Table 5:** Effect of shading nets on mineral accumulation in fruits. Data are expressed as mg 100 g^−1^ fw.

Treatment	P	K	Mg	Na	Malate	Citrate
Control	14.88 ± 0.94 a	412.08 ± 4.76 b	13.04 ± 0.53	6.84 ± 0.72	34.15 ± 1.91 a	140.36 ± 7.84 a
White net	7.63 ± 0.41 b	445.65 ± 1.08 a	12.49 ± 0.43	5.29 ± 0.17	26.78 ± 0.74 b	109.90 ± 1.20 b
Pearl grey net	9.21 ± 0.15 b	361.43 ± 3.58 c	11.23 ± 0.30	6.14 ± 0.32	29.89 ± 1.02 ab	104.33 ± 2.92 b
Significance	**	***	ns	ns	*	**

ns, *, **, and *** non-significant or significant at *p* ≤ 0.05, 0.01, and 0.001, respectively. Different letters within each column indicate significant differences according to Tukey’s HSD test (*p* = 0.05). All data are expressed as mean ± standard error, *n* = 3.

**Table 6:** Effect of shading nets on lutein, lycopene, *β*-carotene, and total carotenoids accumulation in fruits. Data are expressed as mg 100 g^−1^ fw.

Treatment	Lutein	Lycopene	*β*-Carotene	Total Carotenoids
Control	0.022 ± 0.001 b	1.666 ± 0.061 b	0.358 ± 0.012 b	2.046 ± 0.074 b
White net	0.024 ± 0.000 ab	2.881 ± 0.053 a	0.623 ± 0.013 a	3.528 ± 0.065 a
Pearl grey net	0.027 ± 0.001 a	2.828 ± 0.080 a	0.643 ± 0.018 a	3.498 ± 0.099 a
Significance	*	***	***	***

* and *** significant at *p* ≤ 0.05 and 0.001. Different letters within each column indicate significant differences according to Tukey’s HSD test (*p* = 0.05). All data are expressed as mean ± standard error, *n* = 3.

**Table 7:** Effect of shading nets on phenolic compounds accumulation in fruits. Data are expressed as µg 100 g^−1^ fw.

Phenolic Compounds		Treatment		Significance
	Control	White Net	Pearl Grey Net	
PHENOLIC ACID DERIVATIVES				
Chlorogenic acid	3363 ± 105 a	2470 ± 47 b	1799 ± 46 c	***
Homovanillic acid-O-hexoside	939 ± 38 b	1,151 ± 24 a	956 ± 23 b	**
Caffeic acid-O-hexoside	418 ± 19 a	372 ± 5 b	343 ± 11 b	**
Coumaric acid-O-hexoside	74 ± 5 a	56 ± 1 b	81 ± 2 a	**
Ferulic acid	20 ± 0 c	48 ± 2 b	61 ± 3 a	***
Ferulic acid-O-hexoside	19 ± 1 b	34 ± 2 a	21 ± 2 b	*
Caffeic acid	16 ± 1 b	27 ± 1 a	25 ± 1 a	**
Total phenolic acid derivatives	4848 ± 164 a	4157 ± 80 b	3287 ± 78 c	***
FLAVONOID DERIVATIVES				
Rutin	2944 ± 101 b	2481 ± 48 c	4414 ± 112 a	***
Kampferol-3-diglucoside	1979 ± 82 b	1578 ± 24 c	3245 ± 80 a	***
Naringenin	1199 ± 50 b	450 ± 15 c	1851 ± 51 a	***
Rutin-O-pentoside	333 ± 11 b	387 ± 4 b	732 ± 20 a	***
Rutin-O-hexoside	167 ± 6 a	161 ± 2 a	140 ± 6 b	*
Kaempferol-3-O-rutinoside	105 ± 5 c	164 ± 10 b	225 ± 4 a	***
Naringenin-C-diglycoside	72 ± 6 b	78 ± 4 b	225 ± 10 a	***
Apigenin-C-hexoside-hexoside	34 ± 1 c	57 ± 2 b	84 ± 1 a	***
Naringenin-C-hexoside	37 ± 1 b	30 ± 1 c	66 ± 1 a	***
Quercetin-O-dihexoside	14 ± 0 b	12 ± 0 c	21 ± 1 a	***
Genistin	10 ± 0 b	10 ± 1 b	21 ± 0 a	***
Total flavonoid derivatives	6896 ± 263 b	5407 ± 107 c	11,025 ± 276 a	***
HYDROXYCINNAMOYLQUINIC ACID DERIVATIVES				
Dicaffeoylquinic Acid	505 ± 28 a	266 ± 3 b	557 ± 22 a	***
Tricaffeoylquinic Acid	128 ± 7 a	39 ± 3 b	128 ± 5 a	***
Total hydroxycinnamoyl quinic acid derivatives	633 ± 35 a	305 ± 5 b	685 ± 26 a	***
Total phenolic compounds	12,377 ± 460 b	9869 ± 183 c	14,997 ± 378 a	***

*, **, and *** non-significant or significant at *p* ≤ 0.05, 0.01, and 0.001, respectively. Different letters within each column indicate significant differences according to Tukey’s HSD test (*p* = 0.05). All data are expressed as mean ± standard error, *n* = 3.

**Table 8:** Effect of shading nets on DPPH, ABTS, and FRAP antioxidant activities. Data are expressed as mmol Trolox equivalents kg^−1^ dw.

Treatment	DPPH	ABTS	FRAP
Control	32.21 ± 0.40 c	39.18 ± 0.09 b	27.51 ± 0.31 b
White net	35.54 ± 0.37 b	35.33 ± 0.30 c	27.64 ± 0.17 b
Pearl grey net	40.72 ± 0.22 a	43.70 ± 0.58 a	34.38 ± 0.81 a
Significance	***	***	***

*** significant at *p* ≤ 0.001. Different letters within each column indicate significant differences according to Tukey’s HSD test (*p* = 0.05). All data are expressed as mean ± standard error, *n* = 3.

## Data Availability

The data is contained within the article.
